# The Medical and Surgical Treatment of Bright’s Disease1Read before the Section on Medicine, Buffalo Academy of Medicine, May 13, 1903.

**Published:** 1903-08

**Authors:** Charles Sherman Jewett

**Affiliations:** Buffalo, N. Y.


### The Medical and Surgical Treatment of Bright's Disease.[1]

BY CHARLES SHERMAN JEWETT, M.D., Buffalo, N.Y.

THE innumerable attempts to classify satisfactorily the various pathological conditions, which together are known as Bright's disease, have tended toward hopeless confusion rather than toward simplification of our ideas upon this subject. For the purposes of this paper, in the interest of brevity and clearness, and to avoid tiresome repetition, I shall omit all consideration of acute and chronic congestion, acute and chronic degeneration and amyloid degeneration of the kidney, and consider the topic under three general heads:

1. Acute nephritis.
2. Chronic diffuse nephritis with exudation, (chronic desquamative nephritis, chronic tubal nephritis or chronic parenchymatous nephritis).
3. Chronic diffuse nephritis without exudation, (chronic interstitial nephritis, cirrhosis of the kidney, contracted kidney, granular kidney).

In the treatment of acute nephritis we should aim to relieve the congestion and inflammation of the kidneys, to rest these organs as regards their excretory functions, to keep the tubules free but without irritation of the kidney structure, and to meet symptoms as they arise. At the onset of acute nephritis, the patient should be put to bed in a warm room (70 degrees to 74 degrees F.); he should have flannel or canton flannel next the skin, and should lie between woolen blankets. The diet should be liquid, and small in amount, while it may be advisable to withhold all food for the first day or two. In this form of Bright's disease it seems to be universally agreed that an absolute milk diet is best, except only in those cases where milk causes digestive disturbances. If these cannot be otherwise relieved and dilution with lime-water or the use of skim milk fails, we may be forced to the use of weak gruels of arrow-root or barley, or of beef tea, or of chicken broth. It is well to continue the absolute milk diet well into convalescence after both albumin and casts have disappeared from the urine. From the outset large quantities of water or of some alkaline mineral water should be given, and it is best to administer them hot.

For the relief of renal congestion purges, diaphoretic measures, and local applications are to be employed. Hydragogue cathartics and salines are to be given at once and the bowels should be kept active throughout the disease. To promote sweating we may

1. Read before the Section on Medicine, Buffalo Academy of Medicine, May 13, 1903.

use the hot tub bath, the hot air bath, a combination of both, or the hot wet pack. While using these measures hot drinks may be given freely. Should all these means fail, we must consider the advisability of giving pilocarpin subcutaneously. This drug is to be given only with extreme caution and its effects carefully watched. It may be administered with strychnine or digitalis to counteract its depressant action. The sweating induced by pilocarpin may be increased and prolonged by hydrotherapy. The sweating process should be repeated as often as the strength of the patient will permit.

Locally, we may apply hot fomentations or poultices to the loins, covering an area far wider than that occupied by the kidneys. In more severe conditions dry cups, leeches, or wet cups, may be used. Julia, a French author, has reported good results from the local use of an ointment of pilocarpin. High enemata of physiological salt solution at a temperature of 110 degrees F., sometimes act well in relieving renal congestion and promoting diuresis. When there is severe lumbar pain great relief with improvement of all symptoms is said to follow puncture of the kidney. Authorities differ radically as to the use of diuretics in the early stage of this disease, some holding that by relaxing the renal vessels they aggrevate the congestion, while others claim that alkaline diuretics, by rendering the urine alkaline, produce solution and loosening of casts and coagulated matter, and thus free the renal tubules from obstruction. Certain it is that water is our best and safest diuretic.

For nausea and vomiting, occurring early in the disease, we should restrict the ingestion of food, and use ice, minute and frequent doses of dilute hydrocyanic or hydrochloric acid, creosote, carbolic acid, tincture of iodine, or the preparations of bismuth. When these symptoms occur at a later stage of the disease they are due to uremia and should be treated by removing their cause. If uremia should develop with headache, restlessness and sleeplessness, the measures already detailed for renal congestion are to be applied, but with increased vigor,—purges, compound jalap powder, croton oil or elaterium, to produce purgation and free diaphoresis. For the high arterial tension we may use chloral, nitroglycerine, sodium nitrite, and possibly morphine. If convulsions supervene they may be controlled by chloroform, while large doses of potassium bromide and chloral are given per rectum, in the hope of preventing their recurrence. In robust, full-blooded patients phlebotomy with the withdrawal of from one to two pints of blood may be of great service.

Regarding the use of morphine to control uremic convulsions, there is a wide divergence of opinion, it being argued on the one hand that by giving morphine we hinder the elimination of the poison which has caused the convulsions, that we add another poison and that we seriously increase the danger of prolonged and fatal coma. On the other hand it is held that the real danger in convulsions is from the nervous explosion; that where chloroform, chloral and bromides fail, morphine secures rest for the nervous system, gives time for the operation of eliminatives and, finally, that in practice it works well. Osler says that it is indispensable, and he has never noted any bad effects nor any tendency to coma from its use. For the headache and sleeplessness of uremia hyoscine hydrobromate or sulphate, in doses of from 1-200 to 1-100 of a grain, is often of distinct value.

For the dropsy we must use the eliminative measures already detailed to which, after the subsidence of acute symptoms, may be added diuretics. In those cases in which the arterial tension is low or the heart action feeble digitalis, strophanthus, and caffeine are efficient diuretics. When these measures prove ineffective it may be necessary to tap the abdominal or pleural cavities, and this should be done whenever the accumulation of fluid interferes seriously with the respiratory or circulatory functions. Puncture or incision of the skin or the introduction of canulæ into the subcutaneous tissue may in rare cases become necessary to relieve edema, but these operations should be attended with every possible precaution to prevent infection of the wounds. Edema of the glottis or larynx may be treated by steam inhalations, intubation or tracheotomy in case general measures have failed. For cardiac dilatation we may give digitalis, strophanthus, and strychnine. Inflammatory complications should be treated upon general principles.

During convalescence mild preparations of iron and bitter tonics should be given to correct the anemia. The return to full diet should be most gradual and cautious, light farinaceous foods being first allowed, then simple vegetables and fruits, white meats and eggs. Removal to a warm, dry climate is of great value. Chilling of the body surface must be most carefully avoided, and for a long time after apparent recovery occasional examinations of the urine should be made.

### CHRONIC DIFFUSE NEPHRITIS WITH EXUDATION.

In this form of Bright's disease the treatment is very similar to that of acute nephritis. If malaria, syphilis, or chronic suppuration is operative as a cause, that cause should be attacked by appropriate measures and with the hope of securing marked improvement. We should also seek to prevent the further operation of such causes as are to be found in the patient's surroundings and habits of life. Thus, withdrawal from business, the forbidding of severe exercise, regulation of diet, inhibition of alcohol, and the choice of a suitable climate, all have their value in appropriate cases.

The diet should be light and simple,—milk and nonnitrogenous foods, and milk exclusively when there is much albuminuria. The bowels should be kept freely active. Occasional doses of calomel seem to be of much value, not only for their cathartic action, but also as promoting diuresis and absorption of the renal exudate through the lymphatics. The skin should receive peculiar care, and woolen underwear should be worn constantly. Frequent hot baths promote cutaneous elimination and other hydrotherapeutic measures are to be used as indicated, while chilling of the surface is to be sedulously avoided.

Among diuretics water is again the stand-by. Strontium lactate is claimed to possess peculiar efficacy and, in selected cases, digitalis, strophanthus, and caffeine are undoubtedly valuable. Anemia is to be treated by the administration of iron. Occasional changes in the preparation used are advisable. The inhalation of oxygen is said to promote metabolism.

High arterial tension is to be opposed by nitroglycerine, while feeble heart action calls for the exhibition of cardiac stimulants. Chronic uremia is best treated by the vigorous use of diaphoretics and diuretics. Where the financial circumstances and the strength of the patient will permit, a permanent or at least a winter residence in a warm, dry climate, such as that of Arizona, or of Southern California, is often advisable and may tend greatly to the prolongation of life and of comfort. For the acute exacerbations and complications the treatment is in all respects that detailed in considering acute nephritis, although the difference of prognosis in the two conditions is ever to be borne in mind.

### CHRONIC DIFFUSE NEPHRITIS WITHOUT EXUDATION.

Whenever there is reason to believe that lead, or the poisons of syphilis, malaria or gout, stand in a causal relation to this condition, these poisons should be eliminated or counteracted by appropriate treatment. When no such cause is discoverable our treatment can have but two aims; first, to relieve symptoms, and second, to avert or postpone fatal complications, as uremia, apoplexy and heart exhaustion.

Many cases presenting no local symptoms will call for no medicinal treatment. For this class of patients judicious regulation of the habits of life and proper hygiene are all important. Whenever possible relief from mental strain should be secured. Sudden or severe muscular exertion is to be avoided, while gentle and regular exercise is of value to promote a healthy circulation and metabolism. Walking is probably the most desirable form of exercise. It is to be remembered that a moderate increase of vascular tension is necessary and conservative in these cases. Too low tension is probably even more dangerous than excessively high tension. The patients should be warmly clothed, should invariably wear woolen underclothing and, when possible, should live in a dry, warm, equable climate, such as that of Southern California, Arizona, Egypt, or Algeria. Sea voyages are often of value.

The diet should be light and abstemious. A strict milk diet is too weakening and, except in acute exacerbations, is usually inadvisable. Yet milk may well form the basis of diet, combined with the simpler watery vegetables, fruits, and farinaceous foods. Cream, butter and other fats are desirable, and meats are commonly allowed, but in great moderation. Concerning the administration of meats, we encounter wide differences of opinion, from the dictum of Benjamin Ward Richardson, that a pure or nearly pure vegetable diet is best, to the old rule, "one meal of flesh, one of fish, and one of neither." The recent discussion before the American Medical Association seemed to show a modern trend toward the more liberal allowance of meat to these patients. Perhaps the amount of uric acid formation may serve as a reliable guide in determining this point for any individual case. Water or the pleasant alkaline mineral waters should be given most liberally.

The condition of the skin should receive careful attention. Cold bathing is to be avoided, but periodical sweating by means of hot baths, hot air, or Turkish baths, is indispensable. The bowels are to be kept freely active, and this can best be done by the use of salines, or of laxative alkaline mineral waters. Renal activity is to be maintained and every seven to ten days the urine of twenty-four hours should be examined, especially with regard to its acidity and to the elimination of urea and uric acid. Excessive acidity may be corrected by small doses of potassium tartrate, or of the potassio-tartrate of sodium. When symptoms begin to develop they are to be dealt with in much the same way as in the other forms of Bright's disease.

Digestive disturbances are treated upon general principles, except when due to uremia, in which case they are only to be relieved through removing their cause. Excessively high arterial tension is to be diminished by the use of nitroglycerine, nitrites, and the iodide of potassium. The last-named drug, while producing less relaxation of the arterioles, has a more lasting effect than nitroglycerine; hence, often may be given continuously for long periods without disturbance. Where this is not well borne and where nitroglycerine causes headache, tincture of aconite or of veratrum viride, with sweet spirits of nitre, may prove satisfactory. In cases with feeble or dilated heart and low arterial tension digitalis, strophanthus or strychnine is indicated.

For anemia it is customary to give iron preparations, although Tyson claims that in cases of contracted kidney they do far more harm than good. Wier Mitchell, on the other hand, gives from one-half to one dram of the tincture of the perchloride three times daily, holding that this large dosage is of value, both for the anemia and to diminish arterial tension.

Dropsy is to be treated by vigorous purging, sweating, and diuresis,—the alkaline diuretics being here of undeniable value. Eventually, tapping of the great cavities of the body will often be found necessary, but acupuncture and incisions of the skin, as heretofore mentioned, should, if possible, be avoided on account of the great danger of infection.

When uremia threatens we may use purges, diaphoretics, diuretics and nitroglycerine. The withdrawal of twelve to twenty ounces of blood, with or without a subsequent infusion of physiological salt solution, will often be followed by satisfactory results. If uremic convulsions occur we may give inhalations of chloroform, and administer chloral by mouth or by rectum. Croton oil is probably the best purge in this condition. Recurrence is to be guarded against by the use of purges, sweating, diuretics, bromides and chloral. For uremic coma we must use vigorous purging, diaphoresis and blood-letting.

When in spite of the use of vasodilators, troublesome headache, restlessness and insomnia occur, they may at times be relieved by bromides, chloral, sulphonal or hyoscine. These measures failing, morphine is our only resource and, in spite of theoretical objections, its administration often seems justifiable and wise. In the terminal stages, with feeble heart action, relaxed vascular tension, deficient renal excretion and increasing dropsy, we must fall back upon cardiac stimulants and diuretics.

### SURGICAL TREATMENT.

Today no discussion of the therapy of Bright's disease which fails to consider its operative treatment can be regarded as complete. I, therefore, add a brief review of the history of this treatment. In *The Lancet,* January 4, 1896, Reginald Harrison reported three cases upon which he had operated for the relief of supposed surgical affections of the kidney. In each case the only lesion found was an acute or subacute nephritis, and each was followed by complete recovery. Each operation was unilateral and Harrison concluded that operation upon one kidney benefited the functional condition of the other.

In the *British Medical Journal,* October 17, of the same year, Harrison proposed incision of the kidney as a treatment in acute suppression of urine, and in acute nephritis with tenderness on pressure over the kidney, with slow disappearance of casts and albumin. In April, 1899, Edebohls reported six cases of nephropexy upon movable kidneys, the seat of chronic nephritis. Favorable results upon the nephritis in cases 1, 4 and 5, led him to undertake the operation in case 6 for the deliberate purpose of favorably influencing or curing the nephritis. He concludes his paper as follows: "My own favorable experience warrants me, for the present, in regarding chronic nephritis affecting a movable kidney as an important indication for nephropexy."

In June, 1899, Ferguson reported two operations, one was for supposed stone in the kidney, but the pathological report showed chronic interstitial nephritis. Decapsulation was followed by cure. The second case was characterised by chronic pain over the right kidney, with acute exacerbations, but without albuminuria. Septic kidney was suspected but not found. Decapsulation was followed by relief of pain. In this paper Ferguson proposed operation for chronic nephritis.

Israel, June 12, 1899, reported fourteen operations upon the kidney, for the treatment of nephralgia or renal colic and of renal hematuria. Of these cases, three died from nephritis, two were failures, in three the symptoms recurred after long intervals, and six completely recovered. In January, 1902, Israel made the following statement regarding his operations:

I have never attempted to cure with the knife, any form of nephritis whatsoever. On the contrary, my intervention had for its sole object the removal of the distressing or dangerous symptoms of colic and profuse hemorrhages arising in connection with nephritis.

Pousson, October 21, 1899, reported three personal cases of operation upon the kidneys in nephritis, and collected 22 cases from the literature. Edebohls, May 4, 1901, published a further paper in which he said:

I am now prepared to go a step farther and propose surgical intervention for the purpose of attempting a cure of chronic nephritis, whether the affected kidney be movable or in place. When the inflamed kidney is not movable, and operative fixation of the organ is, therefore, not indicated, I shall content myself with entirely denuding the kidney of its capsule proper, and thus affording free opportunity for the formation of new vascular connections, on a large scale, between the bloodvessels of the kidney and those of its fatty capsule.

Harrison, October 19, 1901, reported three additional cases, one of nephrotomy for pain in acute nephritis, one of renal puncture for suppression in acute congestion, and one of incision and drainage for cystic degeneration. Edebohls, December 21, 1901, reported in detail his operations upon nineteen cases of nephritis without mortality. Of these nineteen cases, three disappeared from observation within four to six weeks. In one case (of unilateral nephropexy) no cure resulted. Three years later the opposite kidney was removed, after which the patient lived for five years with only the operated kidney, and died at the end of that time, after undergoing a hysterectomy. Seven cases had been operated upon within less than a year preceding this report and judgment upon them was suspended. The remaining eight cases are reported as cures, having been under observation for periods varying from one year to eight years and four months subsequent to the operation. In seven of these cases the time from operation to the complete disappearance of albumin and casts from the urine, varied from one to twelve months and in one case it was not definitely determined.

In March, 1902, Primrose reported a case of chronic nephritis with albuminuria, hyaline, granular, fatty and epithelial casts, edema, anasarca and ascites. Paracentesis abdominis had been performed nine times. November 21, 1901, the capsule of the right kidney was incised. This was followed by an increased excretion of urine with a lessened percentage of albumin. December 20, 1901, decapsulation of the left kidney was performed. After recovery from a pneumonia, which followed, great improvement in the patient's general condition was observed. Sixty-two days after the second operation the urine contained only a trace of albumin with a few casts, the anemia had nearly disappeared and there was no return of the edema or ascites.

Guiteras, May 17, 1902, reported two operations performed respectively one month and two weeks previously, but he wisely refrains from drawing any deductions from them. In a personal communication dated February 14, 1903, Dr. Guiteras says: "As yet I have drawn no conclusion." He makes no statement of the subsequent history of his two cases.

Edebohls, March 28, 1903, published a further report of his experience. In addition to the nineteen cases already mentioned, he tells of thirty-two others operated upon during the year 1902. These new cases were all far advanced and some of them were operated upon only because the operation was demanded by the patient, or by his physician. In this latest communication Edebohls summarises the results of all his operations as follows:

Summary of results of renal decapsulation for chronic Bright's disease in the author's fifty-one cases, embracing forty-seven operations upon both kidneys and four operations on one kidney only; seven patients died within seventeen days after operation. Seven patients died at periods after operation, varying between two months and eight years; the average period of life after operation being one year and eight months. Two patients do not show improvement satisfactory in every respect. Twenty-two patients are in various stages of satisfactory improvement and progress toward health, at periods varying between two months and fifteen months after operation. The urine of several of these is at present free from albumin and casts. They have not, however, passed the probationary period of six months of normal urine, before the expiration of which no patient is entitled to a place on the list of cures.

One patient, after a cure extending over a period of four years, again has chronic Bright's disease. One of her kidneys only was operated upon. Nine patients were cured of chronic Bright's disease, and remain cured at periods after operation, varying from one year and nine months to ten years, the average duration of cure being over four years. Three patients disappeared from observation after leaving hospital, and no trace of them can be found.

Through the courtesy of Dr. Eugene Wasdin of the Marine Hospital service, I am permitted to read his condensed report of a case operated upon by him. This report is of unique interest, as containing an account of what I believe to be the first autopsy following the performance of Edebohls's operation:

### CHRONIC BRIGHT'S DISEASE, ARTERIOSCLEROSIS, AND MITRAL STENOSIS.

W. K., age 32. History of syphilis for twelve years:

Presented abdominal fluid, great cardiac uneasiness, difficult breathing, urine reduced to 200 c.c. in twenty-four hours; sp. gr., 1.008; albumin, 20 per cent. volume. Although the general condition, contraindicated the operation, it was determined to decorticate the kidneys as a symptomatic measure. The right organ was exposed and the capsule reflected from the cortex over two-thirds of the surface, and the flaps sutured to the muscular walls. Venous hemorrhage was quite free for twenty-four hours. During the first twenty-four hours, the urine increased to 1000 c.c.; sp. gr., 1.008. During the next few days the secretion became greatly increased, 3500 c.c. in twenty-four hours. All symptoms, of course, improved. For two months his condition was such that it was hoped no further operative interference would be required, but at this time, the general symptoms recommenced, dizziness, headache, and the like. The urine gradually diminished in quantity to 500 c.c.

The left kidney was then decorticated, the organ being left free. Again the amount of urine at once increased to 2500 c.c., and symptoms improved; but, again, there was gradual failure to secrete and the man died of uremic coma.

Autopsy limited to kidneys. The first organ decorticated; the right, presented a firm fibrous envelope closely investing the kidney and forming a very tough capsule that could be removed only with difficulty, and which was very slightly supplied with bloodvessels. The left organ was in process of the same change, the kidney being again invested with a firm, tough membrane, which appeared to compress the organ. To neither kidney was there any newly formed blood supply through adhesions. Kidneys incised showed a chronic interstitial nephritis; the organs much reduced in size, with a number of retention cysts in cortex.

Results of operation in this case,—no results as a curative measure for chronic Bright's disease. Good results as a measure to relieve symptoms of imminent danger from suppressed urine and uremia. Its immediate result is diuretic, the duration of which depends upon reparative changes about the kidney. Contraindications as a curative measure,—any organic change in the organs, capable of causing death *per se, i.e.,* arteriosclerosis, heart valve disease, and the like. Cases in which it may be expected to succeed curatively,—in uncomplicated parenchymatous nephritis, and glomerulo-nephritis. To relieve suppression, it should be used in all cases.

I am indebted to Dr. Eugene A. Smith for the opportunity of reporting the following case. Miss H—, age 21, teacher, on April 3, 1902, gave following history:

Two years ago she noticed a swelling of both lower eyelids, which was shortly followed by swelling of the feet and legs; and, finally of the entire body. After persisting for two weeks, the swelling disappeared. During this period the patient complained of severe pain in the left side, with occasional pain in the right side. In September, 1900, she was treated in the hospital for cystitis,—apparently with success. Since then she has had many recurrences of the pain, which was of a cutting character and worse at night. In March, 1902, she had a severe attack of vomiting, with intense pain. Such attacks were frequently repeated for two weeks. After their subsidence, she noticed a swelling of both lower lids, which nearly closed both eyes. April 4, she passed 900 c.c. of smoky acid urine. Sp. gr., 1.010; 9.0 gms. urea, abundant albumin, renal epithelium, hyaline and granular casts. April 21, Dr. Smith decapsulated the left kidney. The operation was undertaken on the probable dignosis of kidney stone, but an apparently typical large, white kidney was found, and no stone.

The operation was followed by general improvement, and the passing of a largely increased quantity of urine, but the latter contained a large amount of albumin and a goodly number of casts. Two days after the operation, the quantity of urine rose to 1152 c.c., and from then until May 25, the daily quantity varied between 1330 c.c. and 2224 c.c. On July 20, 1902, the quantity was 1500 c.c.; sp. gr., 1015; reaction acid; urea, 13.5 gms. There was a large quantity of albumin, and also hyaline and granular casts, cylindroids, red blood-cells, and leucocytes.

Although the hospital records are somewhat vague upon this point, Dr. Smith informs me that from the time of operation there was a very marked diminution in the quantity of albumin, and that during the entire subsequent period of observation this quantity never approached that which was constantly present before operation.

On February 25, 1903, Dr. Smith's patient was seen again, and the following notes taken:

No headaches, strength good, appetite good, bowels regular, color better,—rosy. Urine usually in good quantity, but sometimes diminished. Feet and legs a good deal swollen each night, as high as the knees; eyelids sometimes puffy. She does considerable work, but tires easily. Has occasional slight pain over hips, but usually free from pain. She exercises freely in open air, but sometimes does so reluctantly.

An examination of a single specimen of urine shows the following: amber color, alkaline, sp. gr., 1.012; considerable albumin, no sugar. The sediment contains hyaline, granular and epithelial casts. In view of the amelioration of all symptoms which followed this operation, Dr. Smith is considering the advisability of decapsulating the other kidney.

Edebohls's explanation of the beneficial effects of decapsulation upon the nephritic kidney, are based upon his observations in three secondary operations upon kidneys, upon which nephropexy had previously been performed. I quote the following:

At the second operation, upon the same kidney, the following conditions were noted as results of the first operation: first, the formation of strong connective tissue adhesions or bands attaching the kidney to its surroundings. Second, the existence in these connective tissue adhesions or bands, of very large and numerous bloodvessels, running between the kidney and the adjacent tissues. This fact was forcibly brought home to my assistants and myself by the necessity of ligating artery after artery of considerable size, in dividing these adhesions. Third, the predominance in number and size of the newly formed arteries over the newly formed veins. Fourth, the significant fact that in all the arteries the direction of the blood stream was toward the kidney.

The disparity between the conditions here described and those found at the autopsy in Dr. Wasdin's case, is most striking. Three possible explanations of this disparity suggest themselves: first, that Edebohls's secondary operations may have been upon nonnephritic kidneys, in which case we are hardly justified in assuming that the same conditions would necessarily follow the operation for chronic nephritis; second, that after decapsulation for chronic nephritis the anatomical results may vary widely in different cases; third, that in Dr. Wasdin's case the fact that the capsule was reflected but not removed may, in part at least, account for the findings.

The material at hand is as yet insufficient to warrant the formulation of final deductions regarding the operative treatment of Bright's disease. However, in view of the results already attained, I feel that the following tentative conclusions are justifiable.

1. In certain cases of acute nephritis operation may be of use in relieving suppression of urine, pain, or hematuria; it may perhaps hasten recovery and aid in the prevention of chronic nephritis.

2. In hopeless cases of chronic nephritis decapsulation will sometimes relieve distressing symptoms and prolong life.

3. Should the results claimed by Edebohls be duplicated in the practice of various operators, and should the supposed reparative results of his operation be confirmed by future autopsies, then the possibility of operative cure of certain cases of chronic nephritis must be admitted.

892 Main Street.

